# Draft genomes of two Atlantic bay scallop subspecies *Argopecten irradians irradians* and *A. i. concentricus*

**DOI:** 10.1038/s41597-020-0441-7

**Published:** 2020-03-23

**Authors:** Xiao Liu, Chao Li, Min Chen, Bo Liu, Xiaojun Yan, Junhao Ning, Bin Ma, Guilong Liu, Zhaoshan Zhong, Yanglei Jia, Qiong Shi, Chunde Wang

**Affiliations:** 1 School of Fishery, Zhejiang Ocean University, Zhoushan, 316022 China; 2 Marine Science and Engineering College, Qingdao Agricultural University, Qingdao, 266109 China; 3 Yantai Institute of Coastal Zone Research and Center for Ocean Mega-Science, Chinese Academy of Sciences, Yantai, 264003 China; 4 Yantai Spring-Sea Aquaseeds Co., Ltd., Yantai, 264006 China; 5 Key Laboratory of Experimental Marine Biology, Institute of Oceanology, Chinese Academy of Sciences, Qingdao, 266071 China; 6 Shenzhen Key Lab of Marine Genomics, Guangdong Provincial Key Lab of Molecular Breeding in Marine Economic Animals, BGI Academy of Marine Sciences, BGI Marine, BGI, Shenzhen, 518083 China

**Keywords:** DNA sequencing, Next-generation sequencing, Marine biology, Genome

## Abstract

The two subspecies of Atlantic bay scallop (*Argopecten irradians*), *A. i. irradians* and *A. i. concentricus*, are economically important aquacultural species in northern and southern China. Here, we performed the whole-genome sequencing, assembly, and gene annotation and produced draft genomes for both subspecies. In total, 253.17 and 272.97 gigabases (Gb) of raw reads were generated from Illumina Hiseq and PacBio platforms for *A. i. irradians* and *A. i. concentricus*, respectively. Draft genomes of 835.7 Mb and 874.82 Mb were assembled for the two subspecies, accounting for 83.9% and 89.79% of the estimated sizes of their corresponding genomes, respectively. The contig N50 and scaffold N50 were 78.54 kb and 1.53 Mb for the *A. i. irradians* genome, and those for the *A. i. concentricus* genome were 63.73 kb and 1.25 Mb. Moreover, 26,777 and 25,979 protein-coding genes were predicted for *A. i. irradians* and *A. i. concentricus*, respectively. These valuable genome assemblies lay a solid foundation for future theoretical studies and provide guidance for practical scallop breeding.

## Abstract (summary)

**Table:** 

Measurement(s)	DNA • genome • RNA • transcriptome • sequence_assembly • sequence feature annotation
Technology Type(s)	DNA sequencing assay • RNA sequencing • sequence assembly process • sequence annotation
Sample Characteristic - Organism	Argopecten irradians

Machine-accessible metadata file describing the reported data: 10.6084/m9.figshare.11961984

## Background & Summary

Two subspecies of the Atlantic bay scallop, the northern subspecies *Argopecten irradians irradians* (Lamarck, 1819) and the southern subspecies *A. i. concentricus* (Say, 1822), are widely cultured in China. The northern subspecies *A. i. irradians* is mainly cultured in northern waters, while the southern subspecies *A. i. concentricus* is generally cultured in southern waters^1^. Both subspecies were introduced from the USA between the 1980s and 1990s. In general, these bay scallops grow fast but have short life spans (i.e. <24 months)^2^. These two subspecies are morphologically similar, although the ratio of shell width (W) to shell height (H) or shell length (L) of *A. i. concentricus* is remarkably higher than that of *A. i. irradians*—the average W/L ratio of adult *A. i. concentricus* and *A. i. irradians* are 0.59 and 0.45, respectively^3^. Although both subspecies are adapted to their natural habitats with wide temperature ranges, *A. i. irradians* is more tolerant to the cold northern waters but cannot survive in the southern warm waters, whereas *A. i. concentricus* is better adapted to the warm southern waters but stops growing at a temperature of 12 °C or lower.

Successful diallel crossbreeding has been performed between the two subspecies, as well as between the Peruvian scallop (*Argopecten purpuratus*) and both of the two bay scallop subspecies^4,5^. In addition to high fertilization and hatching rates, the resulting F_1_ hybrids exhibited excellent performance in production traits such as growth and survival, indicating a great potential in stock improvement via inter- or intra-specific hybridization between different subspecies or populations. To date, three high-performance strains, ‘Bohai Red’, ‘QN-2’ and ‘QN Orange’, with increases in average whole body weight of over 38% compared to unselected bay scallops, have been selected from the F_1_ hybrids between the Peruvian scallop and *A. i. irradians*^6,7^. Recently, a new strain was obtained by further crossing the ‘Bohai Red’ strain with *A. i. concentricus*. Interestingly, this strain exhibited a better tolerance to high temperature than the ‘Bohai Red’ strain and had a longer life span than *A. i. concentricus* (Zhigang Liu, personal communication). In addition, the selection of a genetically stable strain in bivalves by traditional breeding methods could take six to ten years, but marker-assisted selection based on genomic data can greatly reduce breeding duration.

In addition to its application in breeding, genomic data can also be immediately employed in studies of evolution, adaptation, longevity, gonad development, and sex determination in bivalves^8–11^. To date, several genomes have been sequenced and assembled in bivalves. For example, assembly of the Pacific oyster (*Crassostrea gigas*) genome provided insights into how sessile oysters adapt to adverse environments^8^. Analyses of the Japanese scallop (*Patinopecten yessoensis*) genome, the first sequenced scallop genome, revealed that scallops may have a conserved primitive karyotype close to that of the ancestral bilaterian^9^. Comparison of the genome sequences of a deep-sea mussel (*Bathymodiolus platifrons*) and a shallow-water mussel (*Modiolus philippinarum*) deepened our understanding of how deep-sea organisms adapt to extreme environments^10^.

In a previous study, we have sequenced and assembled the genome of the Peruvian scallop^12^. The genomic data of *Argopecten* scallops and their hybrids will allow us to investigate the evolutionary relationships among *Argopecten* scallop species and subspecies, to study the molecular mechanisms underlying scallop adaptations to diverse habitats and to understand their wide variation in life span as well as the development of male sterile gonads in their F_1_ hybrids.

In this study, we sequenced and assembled the genomes of the two bay scallop subspecies, *A. i. irradians* and *A. i. concentricus*. Together with the genomic data of the Peruvian scallop, the results of our present study and subsequent genome-wide association studies will eventually facilitate the breeding progress in these *Argopecten* scallops.

## Methods

### Sample collection and genomic DNA extraction

**Fig. 1:**
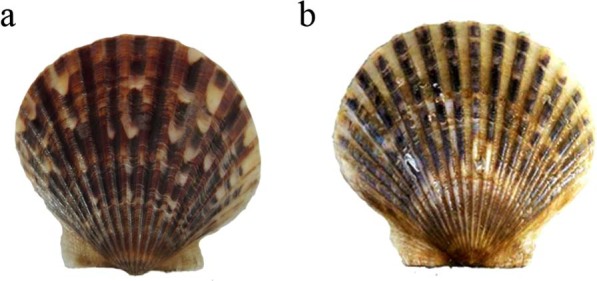
Pictures of the representative bay scallop in China. (**a**) The northern subspecies (*A. i. irradians*). (**b**) The southern subspecies (*A. i. concentricus*).

Genomic DNA was extracted from the adductor muscle of a single specimen from a pure line of *A. i. irradians* (Fig. 1a) and *A. i. concentricus* (Fig. 1b), which have been bred by self-fertilization in a scallop farm in Laizhou, Shandong Province, China. The quality of the DNA samples was checked by electrophoresis on 1% agarose gels. The purity of the DNA was also checked using a NanoPhotometer® spectrophotometer (IMPLEN, CA, USA). DNA concentration was measured using a Qubit® DNA Assay Kit in Qubit® 2.0 Fluorometer (Life Technologies, CA, USA). A total of 1.5 μg DNA per sample was used for subsequent sample preparations.

### DNA library preparation and whole genome sequencing

Sequencing libraries were generated using the Truseq Nano DNA HT Sample Preparation Kit (Illumina, USA) following the manufacturer’s instructions. Index codes were used to cross-index the sequences and samples, that is, the DNA samples were fragmented by sonication and then end-polished, A-tailed, and ligated with the full-length adapter for Illumina sequencing followed by PCR amplification. The resulting PCR products were purified (AMPure XP system) and the sequence libraries were analyzed for size distribution by Agilent2100 Bioanalyzer and quantified using real-time PCR.

These libraries were sequenced on an Illumina HiSeq4000 platform using a 150-bp paired-end sequencing protocol. Additional sequencing was performed on a PacBio Sequel instrument with a Sequel^TM^ Sequencing Kit 1.2.1 (Pacific Biosciences, USA) for both subspecies. Raw reads in the FASTQ format were first processed using Trimmomatic. In this step, clean reads were obtained by removing reads containing adapter sequences, poly-N repeats, and low-quality sequences. In addition, the Q20, Q30, and GC content of the clean reads was determined for quality control. All downstream analyses were based on the high quality, clean reads identified here.

### Genome assembly

To estimate the genome size of both subspecies, a routine 17-mer frequency distribution analysis^13^ was performed according to the following formula: genome size = k-mer number/peak depth (Table 1). A routine assembly strategy was applied for the genome assemblies of both scallops. Briefly, all high-quality reads were assembled into scaffolds using Platanus v1.2.4^14^, and the gaps were filled using GapCloser^15^. Subsequently, PBJelly v14.1 was applied for additional gap filling with Pacbio reads^16^. Finally, all Illumina reads were employed to correct the genome assemblies in Pilon v1.18 for two rounds^17^ (Table 1).

**Table 1:** Summary of the genome assemblies and annotations for both subspecies.

Genome assembly	*A i. irradians*	*A i*. concentricus
Contig N50 size (kb)	78.54	63.73
Scaffold N50 size (Mb)	1.53	1.25
Estimated genome size (Mb)	996.07	974.3
Assembled genome size (Mb)	835.7	874.82
Genome coverage for Illumina reads (×)	254.17	259.6
Genome coverage for Pacbio reads (×)	20.15	20.57
The longest scaffold (bp)	8,652,007	5,002,087
**Genome annotation**	***i. irradians***	***i. concentricus***
Protein-coding gene number	26,777	25,979
Average transcript length (kb)	11.86	12.17
Average CDS length (bp)	1,443.63	1,460.6
Average intron length (bp)	1,704.92	1,722.22
Average exon length (bp)	203.09	202.42
Average exons per gene	7.11	7.22

### Genome assessment

Following the initial assembly, the integrity of both genome assemblies was assessed by mapping the reads from short-insert libraries onto the assembled genomes using Burrows-Wheeler Aligner (BWA)^18^, which can align the clean reads from multiple samples against the reference genomes (settings: bwa mem –t 4 –k 32 –M –R). Alignment files were converted to BAM files using SAMtools^19^. In addition, potential PCR duplications were removed using the SAMtools with command “rmdup”. If multiple read pairs had identical external coordinates, only the pair with the highest mapping quality was retained. Subsequently, the Core Eukaryotic Genes Mapping Approach (CEGMA) was employed to evaluate the completeness of both genome assemblies^20^. Among the 248 ultraconserved core eukaryotic genes (CEGs), we identified 231 (93.15%, complete + partial) and 227 (91.53%, complete + partial) CEGs in the genomes of northern and southern subspecies, respectively. Benchmarking Universal Single-Copy Orthologues (BUSCO) was used to quantitatively assess the completeness of genome assembly based on evolutionarily-informed expectations of gene content from near-universal single-copy orthologues^21^. The assessment demonstrated that 91% of the 843 single-copy genes were identified in both genome assemblies, containing C: 91% [D: 4.3%], F: 6.1%, M: 2.4%, n: 843 (C: complete [D: duplicated], F: fragmented, M: missed, n: groups) for the northern bay scallop subspecies, and containing C: 91% [D: 3.9%], F: 5.3%, M: 3.4%, n: 843 (C: complete [D: duplicated], F: fragmented, M: missed, n: groups) for the southern bay scallop subspecies. These data indicated high integrity of both genome assemblies.

### Repeat annotation

Two methods were employed to identify transposable elements (TEs) in the assembled genomes. When using the *ab-initio* method, RepeatModeler was used to build a species-specific repeat database (parameters set as ‘–engine_db wublast’)^22^. When using a homology-based method, RepeatMasker^23^ was employed to identify repeats with known repeat libraries (Repbase)^24^ using the following parameters: ‘-a -nolow -no_is -norna -parallel 3 -e wublast–pvalue 0.0001’, along with RepeatProteinMask (the parameter set as ‘-noLowSimple -pvalue 0.0001 -engine wublast’)^23^, and the repbase data were collected from a comprehensive database of undifferentiated species (RepBase Metadata Database RELEASE 20170127). In addition, tandem repeats were identified using Tandem Repeats Finder (TRF) with the parameters setting as ‘Match = 2, Mismatching penalty = 7, Delta = 7, PM = 80, PI = 10, Minscore = 50, MaxPeriod = 2,000’^25^ (Table 2).

**Table 2:** Prediction of repeat elements in the two genome assemblies of bay scallop.

Type	Repeat Size (bp)		% of genome	
	*A. i. irradians*	*A. i. concentricus*	*A. i. irradians*	*A. i. concentricus*
TRF	126,153,959	135,900,220	15.10	15.53
RepeatMasker	309,417,572	326,918,089	37.02	37.37
RepeatProteinMask	31,422,581	30,821,540	3.76	3.52
Total	389,681,429	412,788,948	46.63	47.19

### Gene annotation

#### *de novo* prediction

Protein-coding genes in the assembled genomes were annotated using *de novo* prediction by homology with transcriptome data-based evidence. Four programs were employed for the *de novo* prediction of genes, including Augustus v3.2.1 (with the following parameters: ‘-uniqueGeneId true –noInFrameStop = true –gff3 on –genemodel complete –strand both’)^26^, Genscan (using default parameters)^27^, GlimmerHMM (with the following parameters: ‘ -f -g’)^28^, and SNAP (using default parameters)^29^.

#### Homology-based annotation

Protein sequences from mosquito (*Anopheles gambiae*), Amphioxus (*Branchiostoma floridae*), nematode (*Caenorhabditis elegans*), Ascidian (*Ciona intestinalis*), Pacific oyster (*C. gigas*, also known as *Magallana gigas*), fruit fly (*Drosophila melanogaster*), leech (*Helobdella robusta*), human (*Homo sapiens*), owl limpet (*Lottia gigantean*), octopus (*Octopus bimaculoides*), and sea urchin (*Strongylocentrotus purpuratus*) were used for homology-based searches against the two genome assemblies using TBLASTn (e-value ≤ 10^−5^)^30^. The final gene structures were predicted using GeneWise (with the following parameters: ‘-genesf’)^31^.

#### Transcriptome-based annotation

Transcriptome data from different tissues including kidney, hepatopancreas, and haemolymph were mapped onto each genome assembly using Tophat (with the following parameters: ‘–max-intron-length 500000 -m 2–library-type fr-unstranded’)^32^, and used for gene modeling using Cufflinks (with the following parameters: ‘–multi-read-correct’)^33^ according to the pair-end relationships and the overlaps between aligned reads.

#### Gene set integration

Following *de novo* prediction, homology-based annotation, and transcriptome-based prediction, we integrated the gene models using EvidenceModeler (EVM)^30^ to generate a comprehensive and non-redundant gene set (Table 1).

### Functional assignment

Gene function annotation was performed by aligning the predicted protein sequences against various protein databases—including the SwissProt^34^ and NCBI non-redundant (Nr) databases—using BLASTP (e-value ≤ 10^−5^). Gene domain annotation was performed by searching the InterPro database^35^. All genes were aligned against the Kyoto Encyclopedia of Genes and Genomes (KEGG)^36^ database to identify gene pathways. Gene Ontology (GO) terms of the genes were obtained from the corresponding InterPro entry^37^.

### Ortholog and gene family expansion analysis

**Fig. 2:**
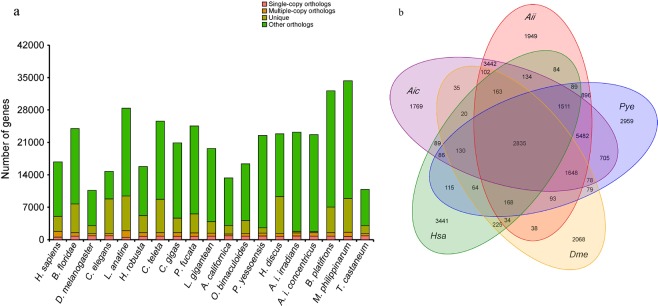
Comparative genome analysis between the bay scallops and the other 19 species. (**a**) Orthologue clustering analysis of the protein-coding genes in the bay scallop genomes. The horizontal axis shows 19 species and the vertical axis shows the corresponding number of genes. Pink represents the number of single-copy gene families, yellow represents the number of multiple-copy gene families, dark yellow represents the number of unique gene families of the corresponding species, and green represents the number of other gene families not mentioned above. (**b**) Venn diagram showing the shared and unique gene families among the five compared species. The total number of each gene family in the unique or shared regions is indicated. Abbreviations of the species are as follow: Aic, *A. i. concentricus*; Aii, *A. i. irradians*; Aca, *A. californica;* Bfl, *B. floridae*; Bpl, *B. platifrons;* Cel, *Caenorhabditis elegans;* Cgi, *C. gigas; Cte, C. teleta;* Dme, *D. melanogaster;* Hdi, *H. discus;* Hro, *H. robusta;* Hsa, *H. sapiens;* Lan, *Lingula anatine;* Lgi, *L. gigantean;* Mph, *M. philippinarum;* Obi, *O. bimaculoides;* Pfu, *P. fucata;* Pye, *P. yessoensis*; Tca, *T. castaneum*.

The protein-coding genes from both scallop genome assemblies and seventeen other sequenced species including Pacific oyster, owl limpet, Amphioxus, nematode, fruit fly, leech, human, octopus, red flour beetle (*Tribolium castaneum*), polychaete (*Capitella teleta*), brachiopod (*Lingula anatina*), sea slug (*Aplysia californica*), abalone (*Haliotis discus*), pearl oyster (*Pinctada fucata*), Yesso scallop (*P. yessoensis*), deep-sea vent mussel (*B. platifrons*) and shallow-water mussel (*M. philippinarum*) were analyzed. All data were downloaded from the Ensembl^38^ or NCBI^39^ databases. Gene family analysis was performed based on the homologs of the protein-coding genes in the related species, which was initially implemented by the alignment of an “all against all” BLASTP. Subsequently, alignments with high-scoring segment pairs (HSPs) were conjoined for each gene pair by Solar^40^ to process the mapping results. To identify homologous gene-pairs, more than 30% coverage of the aligned regions in both homologous genes was required. Finally, homologous genes were clustered into gene families by OrthoMCL^41^. A *p*-value cut-off of 1e-5 was chosen for putative orthologues or paralogs, which were converted into a graph for the nodes of representative protein sequences. The resulting graph is represented by a symmetric similarity matrix to which an MCL algorithm was applied (with the following parameters: “-inflation 1.5”) to regulate cluster tightness (Fig. 2a,b).

### Genome evolution analysis

#### Phylogenetic analysis

To trace the evolutionary position of *A. i. irradians* and *A. i. concentricus*, a dataset containing 107 single-copy protein-coding genes retrieved from the 19 species mentioned above was used for phylogenetic tree construction and divergence time estimation. Protein sequences for these single-copy genes were aligned by MUSCLE^42^ one by one, and then were concatenated to the final dataset. ProtTest^43^ was used to select the best-fit model for this dataset. Then, the phylogenetic tree was reconstructed using the RAxML method (version 7.2.3)^44^ with LG + G + I + F model with the proportion of invariable sites 0.07 and Gamma shape parameter 0.83. The clade containing *H. sapiens and B. floridae* was set as outgroup. Clade support was assessed using the bootstrapping algorithm in the RAxML with 1000 alignment replicates.

#### Estimation of divergence time

Species divergence time was inferred based on the same dataset containing 107 single-copy protein-coding genes from the 19 species using the MCMCTree function included in PAML v4.7a^45^ with the following parameters: ‘–model 0–rootage 1200 -clock 3’. For their divergence time estimation, reference divergence times obtained from TimeTree database^46^ were used as time scales to calibrate the divergence time of *A. i. irradians* and *A. i. concentricus*. These include the divergence times between *T. castaneum* and *D. melanogaster* (307–414 Mya), between *P. yessoensis* and *C. gigas* (>330 Mya), between *C. teleta and D. melanogaster (531–581 Mya), between C. teleta and L. gigantean* (531–581 Mya), between *C. gigas* and *L. gigantean* (500–550 Mya), between *H. robusta* and *C. teleta* (450–602 Mya), between *P. fucata* and *C. gigas* (>330 Mya), and between *B. platifrons* and *M. philippinarum* (39–132 Mya).

## Data Records

The whole genome sequences of *A. i. irradians* and *A. i. concentricus* were deposited in public repositories. The raw sequencing and transcriptomic data were deposited in NCBI Sequence Read Archive, under the SRA study accession SRP174526^47^. This whole-genome project including the assembly fasta, annotation and protein sequencing was uploaded to Dryad (10.5061/dryad.hdr7sqvdr)^48^. All genome annotation and phylogenetic tree files were uploaded to Figshare (10.6084/m9.figshare.c.4356239)^49^. The genome assemblies are also available at the NCBI Assembly website^50,51^.

## Technical Validation

To produce high-quality draft genome assemblies, we applied whole-genome sequencing, assembly, and annotation of the two bay scallop subspecies. The whole genome shotgun sequencing strategy was used for both bay scallop subspecies. We constructed six sequencing libraries including two short-insert libraries (250 bp and 450 bp) and four long-insert libraries (2, 5, 10, and 20 kb) for *A. i. irradians* and *A. i. concentricus*, respectively. For *A. i. irradians*, a total of 253.17 gigabases (Gb) of raw reads were generated while a total of 272.97 Gb of raw reads were generated for *A. i. concentricus*. For *A. i. irradians*, a total of 3.86 × 10^10^ k-mers with a peak k-mer depth of 38 were employed to obtain an estimated genome size of 996.07 Mb (Table 1). In *A. i. concentricus*, a total of 4.97 × 10^10^ k-mers and a peak k-mer depth of 50 were employed to obtain the estimated genome size of 974.3 Mb. Finally, draft genomes of 835.7 Mb and 874.82 Mb were assembled for *A. i. irradians* and *A. i. concentricus*, respectively (Table 1), which accounted for 83.9% and 89.79% of their corresponding estimated genome size (Table 1). For the genome assembly of *A. i. irradians*, the contig N50 was 78.54 kb and the scaffold N50 was 1.53 Mb; meanwhile, the contig and scaffold N50s of the *A. i. concentricus* genome assembly were 63.73 kb and 1.25 Mb, respectively (Table 1). 99.46% of all short reads could be mapped onto the assembled genome of *A. i. irradians* with a coverage of 90.46%. Similarly, in *A. i. concentricus*, 99.4% of all short reads could be mapped onto the assembled genome with a coverage of 86.41%. These mapping results suggest good reliability for both genome assemblies, which are close to the assembly of the Peruvian scallop genome in our previous study but better than those of other related bivalve species^12^.

A protein is classified as complete if the alignment of the predicted protein to the HMM profile represents at least 70% of the original KOG domain, otherwise, it is classified as partial. Our evaluation results demonstrated that both genome assemblies covered 231 (93.15%) of the 248 Core Eukaryotic Gene sequences, indicating a high level of completeness within the two genome assemblies. A related assessment identified 91% of the 843 single-copy genes in both genome assemblies. These data indicate the high integrity of both genome assemblies. A total of 389,681,429 and 412,788,948 bp of repeat sequences were predicted in the *A. i. irradians* and the *A. i. concentricus* genomes, respectively. These repeat sequences accounted for 46.43% and 47.17% of the corresponding genome assemblies (Table 2). A total of 26,777 protein-coding genes were predicted in *A. i. irradians* with an average transcript length of 11.86 kb. The public functional databases Swissport, interpro and NR were used for gene prediction and annotation. Similarly, a total of 25,979 protein-coding genes were predicted in *A. i. concentricus* with an average transcript length of 12.17 kb (Table 1). In total, 24,943 (93.2%) and 24,428 (94%) predicted proteins could be functionally annotated in *A. i. irradians* and *A. i. concentricus*, respectively, using public databases. In total, the protein-coding genes were classified into 48,052 gene families and 107 strict single-copy orthologs (Fig. 2a). Compared to other examined species, 1,949 and 1,769 gene families were exclusively presented in *A. i. irradians* and *A. i. concentricus*, respectively (Fig. 2b).

**Fig. 3:**
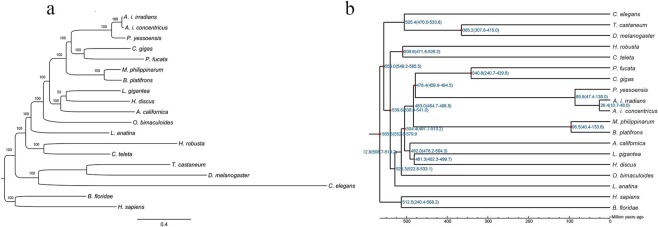
Phylogenetic position of the sequenced species. The phylogenetic tree was constructed based on a dataset from 107 single-copy orthologues using the RAxML method. Clade support was assessed using the bootstrapping algorithm with 1,000 alignment replicates. (**a**) The phylogenetic tree was reconstructed using the RAxML method with LG + G + I + F model. The tree is drawn to scale, with branch lengths proportional to the number of amino acid substitutions. Bootstrap values are presented above the nodes. (**b**) Species divergence time was estimated using the MCMCTree function in the PAML with the parameter of ‘–model 0–rootage 1200 -clock 3’. Red nodes in the phylogenetic tree represented the reference divergence times, which were applied to calibrate the divergence dates of these examined species.

The evolutionary position and divergence time of *A. i. irradians* and *A. i. concentricus* were elucidated in this study. The results of the phylogenetic tree showed that outgroup clade containing *H. sapiens and B. floridae* located in the basal position of the whole tree with high confidence (bootstrap value = 100%). Meanwhile, we found that *A. i. irradians* and *A. i. concentricus* clustered together with the 100% bootstrap value (Fig. 3a), and then merged as a sister group to *P. yessoensis*, as it did in the divergence time tree (Fig. 3b). It shows that this clade has a close relationship with the other two clades containing *C. gigas*, *P. fucata* and *M. philippinarum*, *B. platifrons*. Besides, we estimated the divergence times of *A. i. irradians* and *A. i. concentricus* using single-copy protein-coding genes from the 19 examined species (Fig. 3b). The result showed that the divergence time between the Northern subspecies (*A. i. irradians*) and the Southern subspecies (*A. i. concentricus*) happened at ~26.4 Mya ago, and the analysis suggested that the ancestor of *A. i. irradians*, *A. i. concentricus and P. yessoensis* originated ~85.9 Mya.

## Data Availability

In the study, we did not use any custom specific code. The command line for each step is executed as indicated for each step of all bioinformatics procedures.
